# Practical constraints on estimation of source extent with MEG beamformers

**DOI:** 10.1016/j.neuroimage.2010.10.036

**Published:** 2011-02-14

**Authors:** Arjan Hillebrand, Gareth R. Barnes

**Affiliations:** a VU University Medical Center, Department of Clinical Neurophysiology, Amsterdam, The Netherlands; b Wellcome Trust Centre for Neuroimaging, University College London, London WC1N 3BG, UK

**Keywords:** Spatial extent, Beamforming, Magnetoencephalography, Source modelling, Current density, Neuronal activity

## Abstract

We aimed to determine practical constraints on the estimation of the spatial extent of neuronal activation using MEG beamformers. Correct estimation of spatial extent is a pre-requisite for accurate models of electrical activity, allows one to estimate current density, and enables non-invasive monitoring of functional recovery following stroke.

The output of an MEG beamformer is maximum when the correct source model is used, so that the spatial extent of a source can in principal be determined through evaluation of different source models with the beamformer. Here, we simulated 275-channel MEG data using sources of varying spatial extents that followed the cortical geometry. These data were subsequently used to estimate the spatial extent of generic disc elements without knowledge of the underlying surface, and we compared these results to estimates based on cortical surface geometry (with and without error in surface location).

We found that disc-shaped source models are too simplistic, particularly for areas with high curvature. For areas with low curvature spatial extent was underestimated, although on average there was a linear relationship between the true and estimated extent. In contrast, cortical surface models gave accurate predictions of spatial extent. However, adding small errors (> 2 mm) to the estimated location of the cortical surface abolished this relationship between true and estimated extent, implying that accurate co-registration is needed with such models.

Our results show that models exploiting surface information are necessary in order to model spatial extent and in turn current density, but in order to render such models applicable in practical situations, the accuracy of the cortical surface model itself needs to improve.

## Research Highlights

► Disc-shaped source models are too simplistic, particularly for curved brain regions. ► Cortical surface models give accurate predictions of spatial extent. ► MEG/MRI co-registration accuracy needs to improve to render such models applicable.

## Introduction

It has now been empirically and clinically established that magnetoencephalography (MEG) has the capability to localise changes in electrical activity in the cortex. A challenging problem however is to estimate the extent of electrical activity along the cortical surface. There are a number of reasons why such estimates are important. Primarily, correct estimation of extent of activity is a pre-requisite for an accurate model of the electrical activity. Indeed, sources with a spatial extent exceeding 5 × 5 mm can not be modelled accurately by single equivalent current dipoles (Jerbi et al., 2002). Furthermore, the knowledge of extent would allow one to compute and compare important parameters such as current density, for which at present the best estimates for MEG range from 25 to 400 pAm/mm^2^ (Attal et al., 2007; Lü and Williamson, 1991). Clinically, enlargement of the cortical representation for a specific function seems to be directly related to functional recovery in stroke (Dijkhuizen et al., 2001; Liepert et al., 1998; Nudo et al., 1996; Traversa et al., 1997). Ideally, one would like to monitor these changes in patterns of neuronal activation and improve clinical outcome through therapeutic intervention.

Beamformers are now widely used but are based on simple dipolar models. Vrba has shown that the beamformer output reduces as the true source extent increases, due to the disparity between true and modelled sources (Vrba, 2002). Our motivation for this work was therefore two-fold: to improve the generic beamformer model of the cortical sources, and from this to obtain a measure of spatial extent.

Limpiti and colleagues (2004, 2006) used a limited set of basis functions to describe the activity of a cortical patch, and applied this model to reconstruct sources with either a beamformer or maximum likelihood approach. They showed that their model allows for an accurate reconstruction of extended activity, although they did not quantify the extent of the localised sources. The use of the known cortical geometry has also been exploited in other methods, including Bayesian approaches (Daunizeau et al., 2006; Schmidt et al., 1999), cortical remapping (Baillet et al., 2000; Mosher et al., 1999) and multi-resolution approaches (e.g. Cottereau et al., 2007; David and Garnero, 2002), and methods that model both the spatial properties and temporal dynamics of neuronal populations (Cosandier-Rimele et al., 2007; Jirsa et al., 2002). However, all these methods rely on accurate co-registration of MEG and MRI data. Whalen et al. (2008) report fiducial based co-registration errors of the order of 8.7 mm, which can potentially be improved by surface matching techniques down to 4.4 mm. This is in broad agreement with the 5 mm co-registration accuracy achieved using a dental mold (Adjamian et al., 2004). Assuming that the MRI scan is veridical in size a reasonable bound for the co-registration error in a typical MEG recording is therefore between 5 and 10 mm. Such co-registration errors are especially destructive for beamformers (Hillebrand and Barnes, 2003), as they are very sensitive to deviations between actual and modelled lead fields.^1^

Another approach to estimate extent of activation, without the use of known cortical geometry, has been to use higher order moments of multi-pole expansions (Jerbi et al., 2002; Nolte and Curio, 2000) or generic disc/line elements (Kincses et al., 1999, 2003; Lütkenhöner et al., 1995; Yetik et al., 2005, 2006). This work advances along similar lines, using beamformers to estimate the spatial extent of generic disc elements without knowledge of the underlying surface. We then compare these results to estimates based on cortical surface geometry (with and without error in surface location).

## Methods

In order to validate the method we started by simulating MEG data using disc-shaped extended sources and used similar disc-shaped sources to compute the lead fields for the beamformer source reconstruction (*disc data with disc sources*). We subsequently simulated data for realistically shaped extended sources and used either disc-shaped models (*surface data with disc sources*), or realistically shaped models with the beamformer (*surface data with realistic sources*).

In order to create the source models, a cortical surface was extracted from a subject's anatomical MRI using Freesurfer (Dale et al., 1999; Fischl et al., 1999). The pial surface was triangulated (containing 204996 triangles with a mean (± standard deviation) distance between vertices of 1.0 ± 0.5 mm), and the surface normal for each vertex was computed. A target source, a theoretical equivalent current dipole (ECD), was placed at a selected vertex, oriented perpendicular to the surface. Disc-shaped sources with radii varying from 0 to 18 mm were created by growing a disc-shaped grid, with spacing of 1 mm, in the direction perpendicular to the orientation of the target source. At each element of this disc-shaped grid an ECD was placed with the same orientation as the target source. Realistically shaped sources were formed by selecting elements on the cortical surface within a given cortical distance, varying from 0 to 18 mm from the target source, as calculated along grid vertices (Dijkstra, 1959). At each selected element an ECD was placed with an orientation perpendicular to the cortical surface.

Each ECD in the extended source was given a 40 Hz sinusoidal activation profile and a uniform current density (dependent on the source amplitude and area) was used across the extended source. A total of 9 different source strengths (*Q* = 0, 2, 4, 6, 8, 10, 20, 50 and 100 nAm) were used for time windows lasting for 200 ms (120 samples). One hundred epochs of MEG data were simulated with a per-channel white noise level of 89 fT rms (10 fT/√Hz, 80 Hz bandwidth). For each source with different spatial extent, a separate dataset was constructed from the simulated data, consisting of 9 temporal windows containing data with varying signal-to-noise ratios (SNRs). SNR was computed as follows: for each time window, the average power over all channels in the 35-45 Hz band was divided by the average noise power over all channels in the same frequency band, where the power of the noise was computed for the time window for which the source amplitude was set to zero. A value of 1 was subsequently subtracted in order to obtain the SNR estimate. Note that with this definition of the SNR a source strength of 0 nAm leads to an SNR of 0.

A 275 channel system (CTF Systems Inc.), synthetic 3rd order sensor configuration (Vrba et al., 1999), and a multisphere head model as volume conductor (Huang et al., 1999) were used for the computations of the lead fields. The locations of the cortical surface, head model, and sensors were based on a real experimental recording session (Perry et al., 2010).

A modification of a nonlinear minimum variance beamformer (SAM, (Robinson and Vrba, 1999)) was used to reconstruct the sources underlying the simulated data (see van Veen et al. (1997) and Appendix A for a detailed description of beamformer fundamentals). The modifications consisted of i) the lead fields used in the beamformer formulation were computed for an extended source model, rather than for a single ECD; ii) for disc-shaped sources the optimum orientation of the disc at a given location was determined using the method described by Sekihara and colleagues (2004), rather than by a time-consuming nonlinear search; iii) for realistically shaped sources the local surface normal was used as the orientation of each element in the extended source.

The optimum location of the estimated source was determined in one of three different ways:

In the case of disc-shaped models

i) the target (correct) location of the source that was used to generate the data was used (*without position optimisation*)
ii) using a nonlinear search to find the position of the modelled extended source that maximised the beamformer output (*with position optimisation*). As an initial guess the centre of the modelled extended source was placed at the target (correct) location

For realistically shaped beamformer models

iii) all cortical elements within 2.5 cm of the target location were considered as possible candidates of the extended source centre (*constrained position optimisation*)

For a given dataset and time window of 200 ms (corresponding to a certain source strength), the radius of the modelled source used with the beamformer was varied from 0 to 20 mm. For each extent of the source model, the beamformer output (or maximum beamformer output in case position optimisation was used) was estimated using the data covariance matrix and beamformer weights for that time window, and the corresponding estimated source location (defined as the position of the centre of the extended source) was stored. The Euclidean distance between the estimated source location and the original target location was subsequently computed, and is referred to as the localisation error. The extent of the modelled source for which the beamformer output was at a maximum for a given time window was used as the estimated source extent, which was compared to the extent of the source that was used to generate the data (the true source extent).

The measure we used as the beamformer output was the pseudo-Z statistic, defined as the estimate of the projected source power normalised by the intrinsic channel-noise projected through the beamformer weights (Robinson and Vrba, 1999).

In order to assess the effect of using erroneous surface geometry, we repeated the simulations with the realistically shaped beamformer models, using a cortical surface that was shifted by 2, 5, or 10 mm in randomly chosen directions.

## Results

### Disc data with disc sources

MEG data were simulated for extended disc-shaped sources at a target location in the left visual cortex, with the orientation at the target location in the tangential direction. Data were generated for 10 different noise realisations. The mean (± std) SNR of the simulated data across different source extents and noise realisations, corresponding to source strengths of 0, 2, 4, 6, 8, 10, 20, 50, and 100nAm, was 0.00 ± 0.00, 0.10 ± 0.01, 0.50 ± 0.02, 1.06 ± 0.05, 1.87 ± 0.05, 2.93 ± 0.05, 11.7 ± 0.2, 73 ± 1, and 291 ± 4, respectively.

**Fig. 1:**
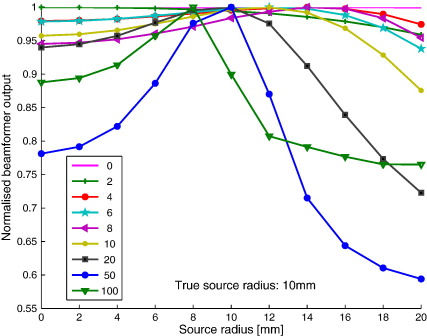
**Normalised beamformer output versus modelled source extent for different SNRs**. Normalised beamformer output was obtained using disc-shaped sources and without position optimisation, using data from disc-shaped sources of varying strength (as depicted in the legend in nAm) and an extent of 10 mm. For a given source strength (SNR), the maximum beamformer output should be obtained when the modelled disc-shaped source has the same radius as the source that generated the data. Note that for low SNRs a change in source extent has little effect on the beamformer output, whereas when SNR increases, the true source extent becomes apparent.

Fig. 1 shows the results for a dataset that was simulated for disc-shaped sources with a radius of 10 mm. Beamformer output peaks when the radius of the modelled disc-shaped source (at the correct location) approaches the true source radius. The maximum is most clearly defined for higher SNRs.

**Fig. 2:**
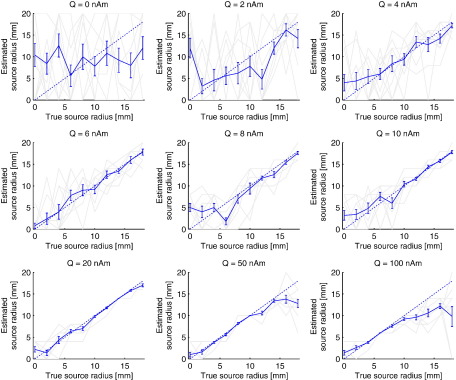
**Estimated source extent versus true source extent for different SNRs**. The same simulation and analysis parameters were used as for Fig. 1. The light grey lines are the results for different noise realisations (N = 10), with the solid blue line the mean and standard error. The dotted line is the ideal result. Note that, on average, source extent is correctly estimated for moderate SNRs. The deviation for large sources at high SNR is due to the orientation selection stage in the beamformer and is discussed below.

**Fig. 3:**
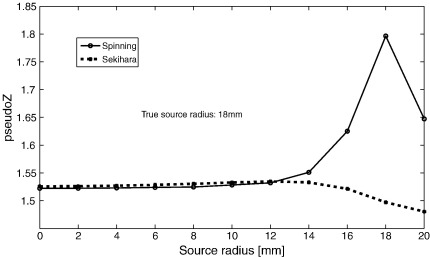
**Comparison of Sekihara's method and nonlinear search approach to determine the optimum source orientation**. The beamformer output was obtained using disc-shaped sources of varying extent and without optimisation for position, using high SNR data (*Q* = 100 nAm) generated by a disc-shaped source with an extent of 18 mm. The optimum orientation of the source was either determined using Sekihara's method, or using a time-consuming nonlinear search for the optimum orientation (‘spinning’). Using the spinning approach the source extent is correctly estimated, whereas with Sekihara's method source extent is incorrectly estimated due to the failure of this approach to find the optimum source orientation for large extended sources (at high SNR).

The effect of SNR and the accuracy of the estimated source extent is quantified in more detail in Fig. 2, where, for different source strengths, the estimated radius (i.e. the radius resulting in maximum beamformer output) is plotted against the true source radius. On average, it is possible to correctly estimate source extent, even for low SNR data (*Q* ≥ 4 nAm), with the accuracy increasing and variability decreasing with increasing SNR. Note that for small extent (< 5 mm) there is a tendency to overestimate the source extent, perhaps because negative source radii are impossible so that estimation errors will lead to positive mean values. Also note that for data with very high SNRs (*Q* = 50 or 100 nAm) and true sources with large extent, there is an underestimation of the source radius. The failure to estimate the true source radius for large, strong, sources seems counterintuitive. Fig. 3 however shows that this is due to the use of Sekihara's approach to find the optimum source orientation. Sekihara's approach is based on the fact that if you know the beamformer output in three orthogonal directions for a point source, then you can compute what the beamformer output will be for a point source at that location pointing in any direction (Sekihara et al., 2004). This is not true for extended sources, since rotating an extended source not only changes the orientation, but also the spatial location, of the elements that form the extended source (except for the element at its centre). This effect is most pronounced when the extent of the underlying source is large, and when the SNR is large enough for this erroneous orientation estimation to be detected (Hillebrand and Barnes, 2003; Limpiti et al., 2006).

### Surface data with disc sources

**Supplementary Figure 1:**
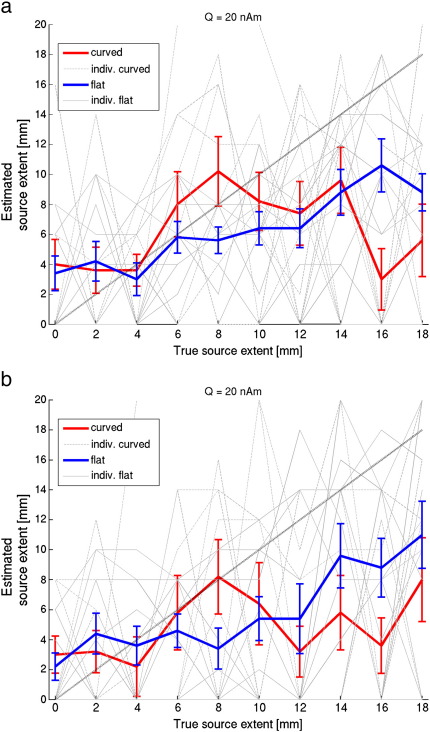
**Estimated source extent versus true source extent using disc-shaped sources with data generated by realistically shaped sources**. Average estimated source extent (solid lines, including standard error) for 10 realistically shaped sources in the left hemisphere with high curvature (‘curved’) and 10 with low curvature (‘flat’). The light grey lines show the curves for the individual sources. The dotted line is the ideal result. The position of the centre of the disc-shaped source model used with the beamformer was either (**a**) optimised, or (**b**) placed at the dame location as the centre of the realistically shaped source that generated the data (no-optimisation). The source strength was 20 nAm. Note the large variability in estimated source extent, as well as the finding that at any single location there is rarely a monotonic linear relationship between true and estimated extent.

**Supplementary Figure 2:**
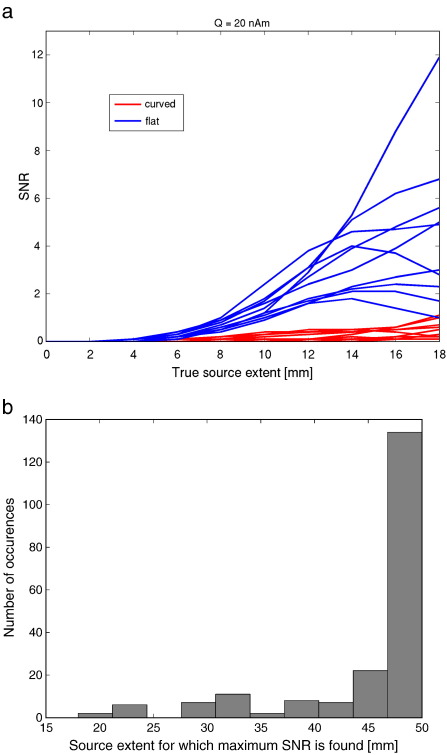
**SNR versus extent for sources with constant current density**. **a)** SNR of data generated by realistically shaped sources of varying extent and with a constant current density of 50pAm/mm^2^ (Lü and Williamson, 1991). Note the large difference in SNR between extended flat and curved sources. Also note that for 60% of the sources the increase in source strength with increasing extent is sufficient to overcome the effect of self-cancellation, resulting in an increase in SNR with extent. For the remaining sources SNR peaked for extents between 10 and 18 mm. **b)** Repeat of the simulations in a) with 200 randomly chosen sources and larger maximum extent. Note that for the majority of sources (63%) the SNR peaks at the maximum simulated source extent.

**Fig. 4:**
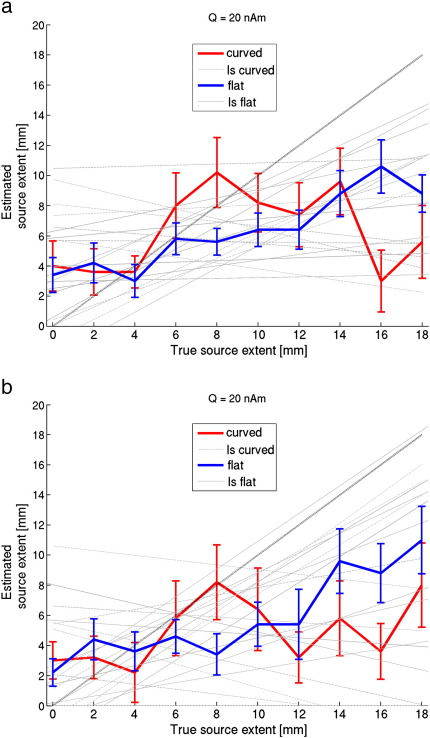
**Estimated source extent versus true source extent using disc-shaped sources with data generated by realistically shaped sources**. Average estimated source extent (solid lines, including standard error) for 10 realistically shaped sources in the left hemisphere with high curvature (‘curved’) and 10 with low curvature (‘flat’). For each source individually, a least-squares fit was performed through the data-points (light grey lines). The dotted line is the ideal result. The position of the centre of the disc-shaped source model used with the beamformer was either (a) optimised, or (b) placed at the same location as the centre of the realistically shaped source that generated the data (no-optimisation). Note the large variability in estimated source extent, as well as the underestimation of source extent for large sources, despite the relatively large source strength of the original source (20 nAm). For the data generated by flat realistically shaped sources the average gradient (± standard deviation) of the regression lines (0.38 ± 0.28) was significantly larger (two-sample *t*(18) = 1.87, *p <* 0.05) than for the curved sources (0.11 ± 0.36) when optimisation was used. With the no-optimisation approach there was no significant difference (two-sample *t*(18) = 1.30 , *p* = 0.11) between the average gradients for the flat (0.44 ± 0.41) and curved sources (0.17 ± 0.50)).

**Fig. 5:**
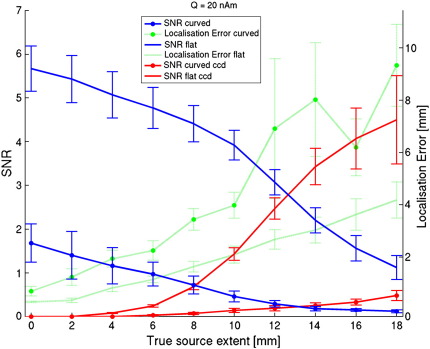
**SNR and localisation error versus extent**. Average SNR of data generated by realistically shaped sources of varying extent (blue lines, including standard error) and average localisation error (green lines, including standard error) obtained when using disc-shaped sources with the beamformer (with position optimisation). Also shown (red lines) is the average SNR for the same realistically shaped sources, but with a constant current density (ccd) of 50 pAm/mm^2^ (Lü and Williamson, 1991). Note the decrease in SNR with increasing source extent due to self-cancellation, and concomitant increase in localisation error for the sources with varying current density. For sources with constant current density the increase in source strength with increasing extent is sufficient to overcome the effect of self-cancellation, resulting in an increase in SNR with extent.

Next, we estimated source extent when disc-shaped sources were used to model data generated by realistically shaped sources. Hence, here we tested whether, given noise in the data, these simple models would still be an accurate enough representation of the local surface geometry. Based on a median split, realistically shaped sources were categorized as ‘curved’ when the average change in orientation across a patch was high (mean ± standard deviation = 19.8 ± 3.7° for 10 sources with extent of 10 mm) and labelled as ‘flat’ when this curvature was low (mean ± standard deviation = 9.0 ± 0.9° for 10 sources with extent of 10 mm). Fig. 4 shows that, again, for smaller sources there is an overestimation of extent. The average (across sources) estimated extent is reasonably accurate for data generated by curved sources with an extent of 4–10 mm. That is, in a between subjects group level analysis one might expect changes in estimated source extent to reflect a real change in active cortical area. However these average (across sources) curves mask a large underlying variability (see also Supplementary Figure 1). The least-squares fits (dotted light grey lines) in Fig. 4a and b demonstrate that for 11/20 individual curved sources there is no positive linear relationship (*p* = 0.41, one-tailed binomial test) between true and estimated extent. In other words, at a single subject level, there is only a 50–50 chance of correctly inferring whether a cortical source has grown in size. At least for data generated by realistically shaped sources with low curvature there is, on average, a significant positive linear relationship between true and estimated extent (F(1,18*)* = 59.1, *p <* 0.0001), although there is an underestimation of extent for all but the smallest sources, and again a large variability (see also Supplementary Figure 1). The substantial underestimation of extent for sources with large extent is due to the drop in SNR caused by self-cancellation of elements within expanding realistically shaped sources. This effect is shown in Fig. 5, where, as expected, self-cancellation is more prominent for curved than for flat realistically shaped sources (blue lines). Fig. 5 further shows that the distance between the centre of the realistically shaped sources and the estimated centre of the disc-shaped source increases when the sources expand. It seems that the model is poor not just because the extent has been incorrectly estimated, but also because the location of the modelled patch is in error. This localisation error increases monotonically with the true source extent and is, predictably, worse over regions of cortex with large curvature. We were also interested in how the sensor level SNR varied as a function of true source extent for sources of constant current density (red lines); here we note that the increasing cortical area (and therefore increasing source strength) gave, on average, rise to a monotonic increase in SNR for both curved and flat sources. This monotonic SNR increase for curved sources was counter to our expectation that we would see a local maximum in SNR due to self-cancellation when a source reaches a certain size. Supplementary Figure 2a shows though that there was considerable variability across sources; for 40% of the sources there was not a monotonic increase in SNR with extent, such that a maximum SNR was found for extents between 10 and 18 mm.

**Fig. 6:**
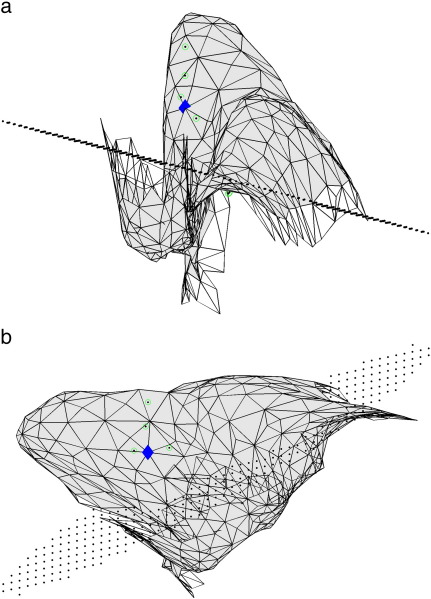
**Realistically shaped extended source with optimised disc-shaped source**. Two different views of the realistically shaped source with extent of 12 mm in left visual cortex that generated the data, together with the grid elements that form the optimum disc-shaped model (radius is 12 mm) as determined by the beamformer. The blue diamond indicates the target location around which the realistically shaped source was grown. The circled dots represent the centre of the disc-shaped source during each step of the position optimisation. Note the poor match between the geometry of the realistically shaped source and the geometry of the disc-shaped source.

Fig. 6 shows an example of how mislocalisation occurs and illustrates that the disc-shaped source is a rather poor approximation for many sections of the cortical sheet. Moreover, for highly curved sections, the centre of the disc shape source, and indeed its radius, bear little relation to the original source that follows the outline of the cortical surface.

### Surface data with realistic sources

**Fig. 7:**
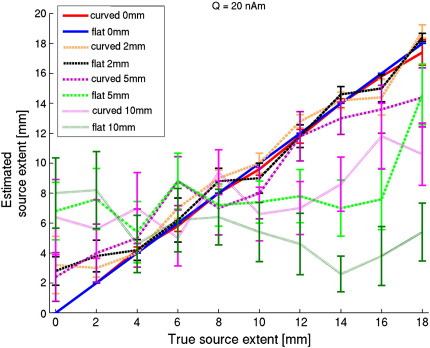
**Estimated source extent versus true source extent using realistically shaped sources with data generated by realistically shaped sources, using correct or incorrect surface geometry**. Average estimated source extent (including standard error) for the same 20 realistically shaped sources as used in Fig. 4. Note the excellent correspondence between the estimated and true source extent, as well as the small variability, when correct surface geometry is used (0 mm shift). When the surface was shifted by 2 mm, the correct extent was still approximated for sources with an extent of 4 mm or larger. For larger shifts the performance severely degraded. Moreover, performance for flat realistically shaped sources was most affected by errors in surface geometry: for flat sources the average gradient (± standard deviation) of the regression lines (not shown) (0.23 ± 0.40 and −0.21 ± 0.51) was significantly smaller than for the curved sources (0.68 ± 0.41 and 0.28 ± 0.45) when the surface was shifted by 5 and 10 mm, respectively (two-sample *t*(18) = −2.49, *p <* 0.05 and *t*(18) = −2.28, *p <* 0.05, respectively). Additionally, for curved sources the relationship between true and estimated source extent was positive and significant for all shifts (*R*^2^ = 1.00, *p <* 1e^−12^; *R*^2^ = 0.97, *p <* 1e^−6^; *R*^2^ = 0.94, *p <* 1e^−5^; *R*^2^ = 0.58, *p <* 0.05 for shifts of 0, 2, 5, and 10 mm, respectively), although the gradient of the average regression line decreased (0.98, 0.88, 0.68 and 0.28 for shifts of 0, 2, 5 and 10 mm, respectively). For flat sources this relationship was only significant (*p* < 1e^−6^) for shifts of 0 and 2 mm.

The extent of the realistic sources could be determined accurately when realistically shaped sources were used to model data generated by realistically shaped sources, as is shown in Fig. 7. The increase in variability with increasing source extent can be explained by the decrease in SNR with increasing source extent (Fig. 5). Note though that despite an average SNR of only 0.62 (across sources) for the largest sources, the true source extent could still be estimated correctly when the correct (i.e. realistically shaped) source model was used. When the cortical surface was shifted by 2 mm, the extent estimations were less accurate, and more variable, with a noticeable overestimation of extent for small sources. Moreover, for shifts of 5 and 10 mm the results degraded further, such that even on average the extent of a source could not be estimated to within standard error bounds. Importantly, the observed large variability reflects the fact that for any given source there rarely (10/20, *p* = 0.59, one-tailed binomial test) was a positive linear relationship between true and estimated extent when the surface was shifted by 10 mm (for shifts of 5 mm a significant number of sources (16/20, *p* < 0.01) still displayed a positive relationship). Interestingly, the effect of using inaccurate surface geometry was more pronounced for the flat realistically shaped sources than for the curved realistically shaped sources, perhaps because of the higher SNR for flat sources, and as a result an increased sensitivity to deviations from the correct source geometry (Hillebrand and Barnes, 2003).

In summary, using realistically shaped sources when the original sources where curved, resulted in, on average, a significant positive relationship between the true and estimated extent, although this relationship weakened with increasing errors in source geometry. When the original sources were flatter, this relationship was only significant when the errors in surface geometry were smaller than 5 mm. Using disc-shaped sources to approximate the curved cortical surface geometry is too simplistic, although on average it is possible to obtain a significant positive linear relationship between true and estimated extent, but only when the curvature is low.

## Discussion and conclusions

In summary, when the cortical surface is known accurately, cortical surface models give accurate predictions of spatial extent, as previously shown by a number of authors (e.g. Baillet et al., 2000; Limpiti et al., 2006, 2004; Schmidt et al., 1999). However, adding a small error (larger than 2 mm) to the grid location abolishes this relationship, implying that without accurate co-registration such models cannot be used to estimate extent. In this study we also assessed the use of simple disc models to estimate source extent. For data generated by flat realistically shaped sources, disc models perform better on average than surface models with errors. Although on average (across locations) a monotonic relationship between true and estimated source extent for disc models is observed, this masks a large underlying variability (and particularly poor performance around curved regions of cortex). We have shown that at any single location there is rarely a linear relationship between true and estimated extent, providing a caveat for experimental designs that predict the growth of a source. Importantly, we should stress that we have examined only the simplest class of extensions of the equivalent current dipole model (which could be termed equivalent current disc/patch models) and then tested how well they could be recovered. Our conclusions are based on the *a-priori* assumption that such source configurations exist, and using such models when the underlying sources have some other current distribution (for example line or sparse non-uniform current distributions) would give erroneous measures of spatial extent.

**Supplementary Figure 3:**
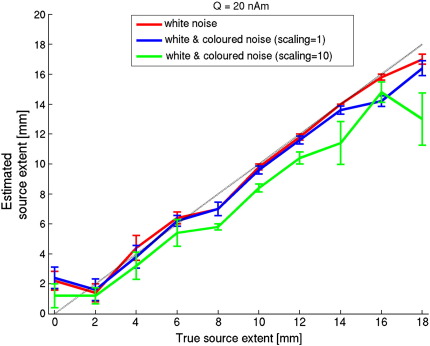
**Estimated source extent versus true source extent for data contaminated by white and coloured noise**. Data was generated using disc-shaped sources with a total source strength of 20 nAm. Normalised beamformer output was obtained using disc-shaped sources and without position optimisation. The curves show the mean and standard error for different noise realisations (*n* = 10), with the noise either being white (red line), or coloured (blue and green lines). Spatially coloured noise was generated by 100 randomly selected cortical dipolar sources, each having a strength of 1nAm and a random activation profile. The generated magnetic field was added to the data generated by the true source and white noise, using a scaling factor of either 1 or 10 for the coloured noise data. Note that the beamformer approach is effective in removing coloured noise (blue line), as has been demonstrated previously (e.g. Adjamian et al., 2009). However, adding large amounts of coloured noise (green line) lowers the SNR, resulting in poorer discrimination of the true source model (see also Fig. 2) and a bias towards underestimation of source extent.

Kincses et al. (1999) reported a general underestimation of extent with realistically shaped models, whereas a modified approach resulted in an overestimation of extent for small sources (Kincses et al., 2003). We also found small overestimation of extent for disc sources with small radii (Fig. 2). Part of this error could be attributed to the fact that negative radii are impossible and therefore any error will increase, and never decrease, the mean estimated extent. A similar ceiling effect may explain the underestimation for large sources (20 mm was the maximum extent used for the model). The underestimation of extent was more pronounced for low to moderate SNRs than for high SNRs. This effect, and the effect of coloured noise (Supplementary Figure 3), could be of importance when comparing data for two groups, such as patients and controls, which may also differ in SNR.

We assumed a uniform activation profile (see also Kincses et al., 1999, 2003). Using a more complicated model for the data generated by disc-shaped sources, one where the activation profile of a patch is determined by the distance between the elements of the patch and its centre (David and Garnero, 2002), gave quantitatively similar results (not shown). Similarly, Limpiti et al. (2006) have shown that with their approach the exact activity profile is not an important factor for extended sources, except for extremely small patches.

We did not test the specific situation where the original sources extended in only one direction (Cao et al., 2006; Yetik et al., 2005), as we don't consider such source models to be very realistic. However, on the basis of our results with realistically shaped original sources, modelled by disc sources (see “*Surface data with disc sources*” in the Results section), we would predict that the mismatch between line sources and disc sources/realistically shaped extended sources would again lead to erroneous estimates of source extent. Moreover, when testing models of different extent, we restricted ourselves to realistically shaped sources models that expanded in a concentric fashion. In practice, the generators of the MEG signal might not have such a symmetric shape, so that one may also need to test with extended cortical patches of arbitrary shape. Similarly, in order to speed up the computations, we modified Sekihara's approach (Sekihara et al., 2004) to find the optimum orientation for disc-shaped sources (Appendix A), even though this approach does not necessarily result in optimum performance (Fig. 3). In practice therefore, when using disc-shaped models, one should use the time-consuming exhaustive search-for-optimum orientation approach (Robinson and Vrba, 1999), or use a vector beamformer.

We did not study the effect of grid density on extent estimates. In practice, the density of the cortical patch should be sufficiently high to ensure that the source model forms an accurate representation of a true cortical source (i.e. that it captures the details of the geometry). The results in Fig. 7 suggest that the variation of lead fields across the smooth cortical surface is such that a spatial sampling of the order of 2 mm is acceptable.

The simulated data in this work was only contaminated with spatially white noise. Empirical MEG data also contain noise that is correlated across channels, and our results should therefore be considered as a best case scenario. By design, beamformers reject coloured noise, hence one would expect that the addition of coloured noise has the same effect as using a beamformer with lower SNR data, i.e. poorer discrimination of the true source model. To our surprise, these is a bias towards underestimation when large amounts of coloured noise are present (Supplementary Figure 3). This bias cannot be due to noise-induced sidelobes in the beamformer images, which could potentially cause biased localisation and erroneous extent estimates, because the source location was correctly estimated. That said, the effect of coloured noise is relatively small compared with that of co-registration errors and we see this as the major methodological hurdle.

Our results clearly show that models exploiting accurate surface information are necessary in order to model spatial extent and in turn current density. The accuracy of this information depends on a number of factors, including accuracy of estimation of fiducial locations and surface matching (Adjamian et al., 2004), as well as MRI distortions. In addition, the choice of cortical surface (outer boundary or inner boundary of the grey matter) adds an uncertainty that is in the range of tolerable geometrical accuracy. Clearly none of these errors are insurmountable, but would need to be addressed. One potential approach, which eschews surface matching and fiducial errors, is to use the anatomical information contained within the MEG functional image to refine co-registration (Barnes et al., 2005; Woods et al., 2008). Limpiti and colleagues (2006) have suggested that using a limited set of basis functions to describe a cortical patch provides a trade-off between modelling accuracy and spatial resolution, and that this may reduce the sensitivity to errors in the forward solution (i.e., MRI segmentation and co-registration errors). Similarly, regularisation of the beamformer (in this work no regularisation has been applied) would lessen the sensitivity to modelling errors but in doing so would also reduce the selectivity to source extent.

One could argue that the gain in SNR by decreasing source extent might mitigate the effects of modelling errors. However, for realistically shaped sources, the loss in performance due to co-registration errors is similar for original sources with different spatial extents (Fig. 7), whereas SNR increases when the source extent decreases (Fig. 5, blue line). Taken together, these results therefore imply that an increase in SNR is not able to compensate for the attenuation due to modelling errors caused by erroneous co-registration.

The main assumption behind the beamformer approach is that each cortical source has a time course that is not linearly correlated with any other source. An extended cortical source comprises many correlated sources, hence a reduction in beamformer performance is to be expected (hence the discrimination between true and incorrect models shown in Fig. 1). However, in this work we explicitly modelled the source correlation (Brookes et al., 2007) and as a consequence the performance did not degrade due to correlations within the extended sources.

Modelling aside, we also looked at sensor level SNR when sources of fixed current density increased in area (Fig. 5). We expected that as source area increased, SNR would continue to increase until the point where the source becomes self-cancelling. We were surprised that this was not the case in our original simulations; SNR continued to increase monotonically with source extent for a majority of sources. To test if this was an artefact due to the small sample size and limited extent, we repeated the simulation in Fig. 5 with 200 randomly chosen sources that were allowed to grow up to 50 mm in radius (see Supplementary Figure 2b). Again, we found that for the majority (63%) of cortical locations the largest SNR is found for the most extended sources. Interestingly, although Ahlfors and colleagues did not examine the effect of source extent on SNR explicitly, their example comparing a small and large extended patch (Fig. 3 and accompanying text in (Ahlfors et al., 2010) also shows that there is a doubling of signal amplitude for an extended source, despite a large amount of self-cancellation. It seems that although along certain dimensions (e.g. a source extending from a sulcus into a gyrus) there is self-cancellation among the elements that form the expanding sources, this effect is mitigated by the SNR increase due to spread along the orthogonal surface dimension (e.g. the extent of the rest of the source along the sulcus). That is, although accurate modelling of an extended realistically shaped source by simple disc-shaped models may be a problem, the detection of that source will in general be facilitated. Moreover, the increased SNR will also facilitate extent estimation with surface-based source models, provided accurate MEG-MRI co-registration and surface extraction (Fig. 7). However, for 37% of the cortical sources (assuming constant current density), the cortical folding acts as a spatial filter by virtue of its curvature, such that MEG is optimally sensitive to sources with radii between 18 and 48 mm (Supplementary Figure 2b), and/or leading to SNR-versus-extent curves (not shown) that contain a local maximum (for 149/200 sources) at extents smaller than 50 mm. Importantly, this behaviour depends on the exact local surface geometry (see also Goldenholz et al., 2009). Again, these results are consistent with the findings by Ahlfors et al., who have shown that, on average, when a source increases in extent there is at first little self-cancellation, followed by a regime where there is large self-cancellation, and finally a large regime where there is almost no further self-cancellation (Ahlfors et al., 2010). In terms of SNR, one would predict that this behaviour leads to an initial increase in SNR, a subsequent drop in SNR, and then an increase in SNR again; depending on the exact cortical geometry this results in an SNR-versus-extent curve with either a local maximum, or one with a maximum SNR for the source with maximum extent.

The non-monotonic variation of SNR with extent means that methods based on the assumption that the dipole moment is directly related to the number of active pyramidal cells (Tecchio et al., 2000) may not always correctly associate a change in dipole moment with an increase or decrease in true source extent (Roberts et al., 2000). Such information linking current density, spatial extent and location could however provide useful constraints on the inverse problem, which could fit naturally into existing Bayesian approaches (Friston et al., 2008; Sato et al., 2004; Yoshioka et al., 2008).

Although our initial concern was modelling error (and hence reduced distorted beamformer output), it is worth noting that typically the use of models with incorrect source extent (such as the commonly used single dipole) has very little effect on the final source image. This is due to the fact that for moderate SNRs the curves of projected power against source extent are relatively shallow (Fig. 1). For example, even for high SNR (20 nAm) a dipolar source still gives rise to 94% of the signal one expects from the optimal model (extended source with 10 mm radius). That is, given the above constraints (co-registration, SNR etc.) the dipolar model for beamformers seems to be more than adequate for most practical situations.

The following are the supplementary materials related to this article.

## Appendix A The basics of beamforming

The output of a beamformer at a location of interest can be defined as the weighted sum of the output of all (N) signal channels (van Veen et al., 1997), or mathematically:

(1) V=W·B,

with *V* the beamformer output (source strength in nAm), **W** the 1xN weight vector (for a given direction of the source at the target location) and **B** the Nx*T*_1_ matrix of the magnetic field at the sensor locations at all (*T*_1_) latencies. Note that the beamformer output at a target location has the same temporal resolution as the recorded MEG signals, and is therefore often referred to as a Virtual Electrode.

The weights determine the spatial filtering characteristics of the beamformer and are designed to increase the sensitivity to signals from a location of interest whilst reducing the contribution of signals from (noise) sources at different locations. The beamformer weights for a source at a location of interest are completely determined by the data covariance matrix and the forward solution (lead field) for the target source (see van Drongelen et al., 1996; van Veen et al., 1997; Robinson and Vrba, 1999; Mosher et al., 2003):

(2) $$V=C_{\text{j}}\;L^{\text{T}}\;C_{\text{b}}^{\text{-1}}\;Β\text{,}$$

with **C**_j_ the source current covariance matrix, **C**_b_ the data covariance matrix, **L** the lead field and T the matrix transpose.

Differences between various source reconstruction algorithms arise from the different assumptions that are made about the source current covariance matrix (see Mosher et al., 2003; Hillebrand et al., 2005). In the case of the beamforming approach it is assumed that all sources are uncorrelated, i.e. **C**_j_ is a diagonal matrix, and that each diagonal element in **C**_j_, corresponding to a location θ, can be related to the measured data as follows (Mosher et al., 2003):

(3) $${\text{σ}}_{\text{θ}}^{2}={\left(L_{\text{θ}}^{\text{T}}\;C_{\text{b}}^{\text{-1}}\;L_{\text{θ}}\right)}^{-1}$$

Eq. (3) is the crux of the beamformer algorithm. It is here that the source covariance **C**_j_ is estimated based on the data.

So far we assumed that the source orientation is known. In practice, one can perform a search for the orientation that optimises the beamformer output (Robinson and Vrba, 1999), compute the beamformer output for the two tangential orientation components (or all 3 orthogonal components in the case of EEG) and obtain the vector sum (van Veen et al., 1997; Sekihara et al., 2001), or using an eigenvector decomposition (Sekihara et al., 2004). In this work, when using disc-shaped models, Sekihara's approach was used, with the only difference with the original description (Sekihara et al., 2004) being that we used the sum of the lead fields for all the elements in the disc-shaped extended source instead of the lead field for a single equivalent current dipole. Note that for extended sources this approach does not necessary result in the optimum source orientation, but that this only leads to unacceptable results if both the SNR and the extent of the sources is large (see Fig. 3 in the main text, and accompanying discussion).

The beamformer output can be computed sequentially for all voxels in a pre-defined source-space, forming a 3-dimensional image of source activity. These images exhibit a non-uniform projection of sensor noise (the weights increase with depth, but the sensor level noise remains constant) throughout the volume (see Robinson and Vrba, 1999). Normalising the beamformer output can compensate for this inherent bias. We will describe the normalisation used by Robinson and Vrba here, although slightly different normalisation approaches are also in use by other beamformer implementations (see Huang et al., 2004 for review). Assume that the sensor noise covariance matrix, **Σ**, is known, then the normalised beamformer output can be computed as (Robinson and Vrba, 1999):

(4) $${\text{Ƶ}}_{\text{θ}}^{2}=\frac{{\text{P}}_{\text{θ}}}{{\text{N}}_{\text{θ}}}=\frac{W_{\text{θ}}^{\text{T}}\;C_{\text{b}}W_{\text{θ}}}{W_{\text{θ}}^{\text{T}}\;\Sigma W_{\text{θ}}}\text{,}$$

with N_θ_ the power of the projected sensor noise and Ƶ_θ_ the pseudo-Z statistic for location θ.

## Footnotes

1 The lead field is defined as the MEG sensor signal that is produced by a source of unitary strength.
